# β-Cyclodextrin Modified Hydrogels of Kappa-Carrageenan for Methotrexate Delivery

**DOI:** 10.3390/pharmaceutics15092244

**Published:** 2023-08-30

**Authors:** Maria Nikitina, Nataliya Kochkina, Marianna Arinina, Valery Kulichikhin, Irina Terekhova

**Affiliations:** 1 G.A. Krestov Institute of Solution Chemistry of RAS, 153045 Ivanovo, Russia; 2 A.V. Topchiev Institute of Petrochemical Synthesis of RAS, 119991 Moscow, Russia

**Keywords:** carrageenan, methotrexate, cyclodextrin, gel

## Abstract

This work is aimed at developing a kappa-carrageenan (kCR) gel with increased methotrexate (MTX) content. β-Cyclodextrin (βCD), which is able to inclusion complex formation with MTX, has been used to increase the drug concentration in the hydrogel. The rheological behavior of the designed gels was investigated and the influence of MTX and βCD on the viscoelastic properties of kCR gel was studied in detail. The effect of βCD and its concentration on the MTX-releasing rate from the kCR gels was examined. The properties of kappa- and iota-carrageenans loaded with MTX were compared and the differences observed were explained in terms of different binding affinities of MTX to these polymers. The obtained gels provided desirable viscoelastic properties useful for topical application.

## 1. Introduction

Pharmacological hydrogels are soft dosage forms which are used to treat a wide variety of diseases. Hydrogels are considered as three-dimensional polymeric networks insoluble in water due to the presence of chemical crosslinks (tie-points, junctions), or physical crosslinks, such as entanglements or crystallites [1]. These crosslinked polymer structures are capable of imbibing large amounts of water or aqueous solutions of biologically and pharmacologically active substances. From a pharmaceutical point of view, these hydrogels must be chemically and biologically safe as well as biocompatible and stable during storage, have specified rheological characteristics, and ensure drug bioavailability. Additionally, one of the modern requirements of hydrogels for biomedical and pharmaceutical usage is the ability to sustain release of the active pharmaceutical ingredient. This property of the gels gives the opportunity to increase the duration of the therapeutic effect and, consequently, to reduce the frequency of medicine application.

**Figure 1:**
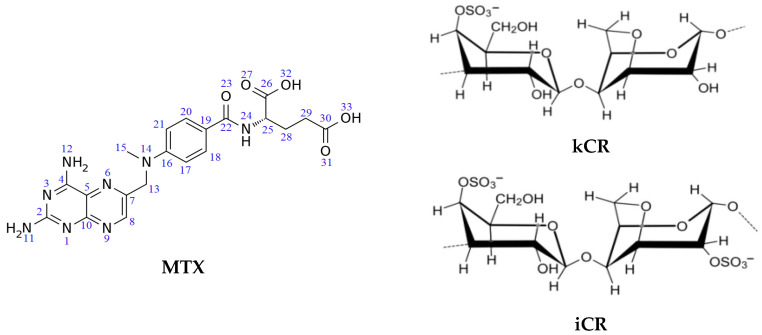
Chemical structure of MTX, kCR and iCR.

Among the polymers capable for gelling, carrageenans (CRs) are natural, non-toxic and biocompatible polysaccharides produced from red seaweeds [2,3]. CRs, being linear sulphated galactans, exist in several different forms based on their sulfate content (Figure 1). Among the CR family, only kappa-carrageenan (kCR) and iota-carrageenan (iCR) possess gelling capability, suitable physicochemical properties, high water-holding capacity, and good adhesion to the skin and mucosal surfaces [3,4]. Moreover, they exhibit antiviral (against coronaviruses, dengue virus, herpes simplex virus, vesicular stomatitis virus, human immunodeficiency virus, influenza virus, human papillomavirus, etc.) and immunomodulatory activity [5,6]. The US Food and Drug Administration has generally recognized kCR and iCR as safe for topical application and consumption [6]. For instance, the efficacy and safety of a CR-based gel to prevent human papillomavirus infection have been recently demonstrated by Laurie et al. [7]. Nasally administered iCR improved outcomes in COVID-19 patients [6]. Furthermore, CR gels can be used for transmucosal and transdermal delivery of biologically active substances, tissue engineering, and regenerative medicine [3,4,5,8,9,10,11,12,13,14,15,16]. For instance, triamcinolone acetonide has been loaded in kCR gel for ocular delivery [8]. Yermak et al. [9] proposed CR gel beads for ophthalmic and oral delivery of echinochrome. Moreover, CR hydrogels have been used to administer acetaminophen orally to patients who have difficulty swallowing conventional formulations such as tablets or capsules [9,10]. There are kCR gels available for the delivery of β-carotene [11], silver nanoparticles [12], metformin hydrochloride [13], zaltoprofen [14], lidocaine [15], and brimonidine tartrate [16]. Hydrogels of CRs have been extensively studied as wound dressing materials because of their high water-holding capacity and biocompatibility [17]. Curcumin hydrogel film based on CR has been developed and proposed as a functional wound dressing material [18]. CR-based hydrogel film reinforced with sulfur nanoparticles and grapefruit seed extract has been designed for wound healing application [19].

Recently, we proposed iCR gels for methotrexate (MTX) delivery [20]. MTX (Figure 1) is a therapeutic agent having a wide range of applications, including treatment of oncological and autoimmune diseases [21]. The topical use of MTX may help to weaken or eliminate a number of serious side effects caused by the oral administration of MTX. Topical gels with MTX have been extensively studied [22,23,24,25]. There are some examples. Carbomer gels bearing MTX have showed improved topical delivery intended for effective management of psoriasis [22,23]. MTX-loaded chitin [24] and chitosan/hyaluronan [25] nanogels have been formulated for its topical use in psoriasis.

This work, being a continuation of our previous study [20], focuses on kCR gels, which for the first time are proposed for MTX delivery. To the best of our knowledge, only one publication [26] concerning MTX-loaded magnetic kCR/chitosan hydrogels has been found in the literature. The MTX encapsulation efficiency in this gel has been increased by increasing its magnetite and chitosan contents [26].

Since MTX is poorly soluble in water [27,28,29], the employment of solubilizing agents is necessary to increase the aqueous solubility and, consequently, the content of MTX in kCR hydrogel. Cyclodextrins (CDs), being native cyclic oligosaccharides, are widely used as solubilizers due to their ability to include guest molecules into a hydrophobic inner cavity and form inclusion (or host–guest) complexes [30,31]. Among native CDs, only βCD exhibits a more pronounced solubilizing effect on MTX [20,32,33]. In this connection, βCD can be used to increase the MTX concentration in the kCR gel. Thus, the purpose of the present work was to design and characterize the kCR gels with MTX and βCD content, and the following aspects were investigated: (i) the influence of MTX (as active pharmaceutical ingredient) and βCD (as solubilizer) on the rheological behavior of kCR gel; (ii) the possible interactions of MTX and βCD with the kCR network; (iii) the release of MTX from the gels and transmembrane permeability in vitro.

It should be also emphasized that the physicochemical and functional properties of CR gels are determined by the polymer structure, and, therefore, for iCR and kCR gels, they can be different. As is known, kCR contains only one sulfate group per disaccharide (Figure 1) and forms rigid gels with high swelling ability. On the contrary, iCR has two sulfate groups per disaccharide-repeating unit (Figure 1) and form more soft gels [1,2]. The carrageenan gels with various rheological characteristics could display different pharmacological properties. Hence, it was interesting to compare the gels of iCR and kCR with MTX. The influence of the number of sulfate groups in the CR structure on the binding affinity to MTX was considered herein.

## 2. Materials and Methods

### 2.1. Materials

kCR, MTX, and βCD were purchased from Sigma-Aldrich (Moscow, Russia) and used without additional purification. The content of cations in kCR was as follows: K^+^ (2.6 wt.%), Na^+^ (6.6 wt.%), and Ca^2+^ (7.7 wt.%). All other chemicals (Na_2_HPO_4_, KH_2_PO_4_, NaOH) were of analytical reagent grade and used without previous purification. Double distilled water was used for gels and buffer solutions preparation. The pH of the solutions was determined by means of Five Easy pH-meter (Mettler Toledo, Columbus, OH, USA) standardized using reference solutions.

### 2.2. Preparation of Hydrogels

The kCR hydrogel was prepared by dissolving kCR powder in distilled water at 80 °C for 20 min under magnetic stirring until the powder was fully swollen and solubilized. Then, the solution was slowly cooled to room temperature.

To obtain kCR/MTX gel, the saturated solution of MTX (2 × 10^−4^ M) was obtained and then used to prepare the gel as described above. To prepare the kCR/βCD gel, βCD (1 wt.%) was preliminarily dissolved in distilled water and then kCR powder was added to this solution. The protocol for kCR/MTX/βCD gel was as follows: βCD solution was prepared and used to obtain a saturated MTX solution, to which, kCR was added. The kCR concentration in all hydrogels under study was 1.25 wt.%. The content of the gels under study is reported in Table S1I. All gels were held for 24 h before tests to let the kCR swell properly and form a homogeneous solution.

### 2.3. Rheological Measurements

The rheological properties of the gels were measured by means of HAAKE MARS 60 Rheometer (Thermo Fisher Scientific, Dreieich, Germany) using a cone-plate (CP) measuring cell (CP 20/1°). All rheological measurements were performed at 25 °C and 37 °C in duplicate. Temperature was controlled with accuracy of 0.1 °C by the Peltier elements.

The gels were kept in the measuring cell for 15 min before the measurements for the temperature stabilization.

At each temperature, the samples were measured in the following regimes:

(1) Oscillation to obtain frequency dependences of the storage and loss moduli in the linear viscoelasticity range. The strain value was 0.5% and the frequency varied from 0.05 to 100 Hz;
(2) Shear rate control mode to obtain flow curves; the shear rate was increased from 0.004 to 5000 s^−1^ in a step-wise mode, with a duration of deformation of 30 s at every shear rate step.

### 2.4. Scanning Electron Microscopy (SEM)

The SEM photos of the freeze-dried gels were made using the Quattro S microscope (Thermo Fisher Scientific), operating at 5 kV.

### 2.5. ^1^H NMR

A Bruker-AV-500 ^1^H NMR spectrometer was used. The constant temperature of 25 °C was maintained with the help of Bruker BVT-3000 temperature controller. Deuterated water (isotopic purity is 99.9%) was used as solvent in these experiments.

### 2.6. FTIR Spectroscopy

FTIR spectra of the freeze-dried gels were acquired using the Fourier transform infrared spectrometer Vertex 80 v (Munich, Germany). Spectra were recorded in the range of 400–4000 cm^−1^. A KBr disc was prepared for each sample.

### 2.7. Dynamic Light Scattering

Particle size distribution in the samples was determined by the dynamic light scattering (DLS) method using a Zetasizer Nano ZS analyzer (Malvern Instruments, Malvern, UK). Each sample was filtered through a 0.45 μm filter and analysed after 24 h of sample preparation. Measurements were performed at 25 °C in the 173-degree backscattering mode. Concentration of the reagents was as follows: 0.01 wt.% for kCR, 2 × 10^−4^ M for MTX, and 1 wt.% for βCD. Each sample was measured three times and the average value was taken.

### 2.8. Release Study

The in vitro release of MTX from the kCR hydrogels without and with βCD was determined in phosphate buffer pH 7.4 (0.04 M Na_2_HPO_4_·12H_2_O, 0.03 M NaH_2_PO_4_·2H_2_O, 0.17 M NaCl) as release medium. The samples were stored in a hermetically sealed cuvette at 37 °C. At each time point, the absorbance of the solution was measured using a UV–vis spectrophotometer (UV-1800, Shimadzu, Tokyo, Japan) at 258 nm. MTX concentration was calculated using a previously obtained calibration curve (Figure S1I).

### 2.9. In Vitro Transmembrane Permeation Study

The in vitro permeation study was performed using a vertical Franz diffusion cell (PermeGear Inc., Hellertown, PA, USA). The receptor compartment was filled with 5 mL of phosphate buffer and stirred at 500 rpm. Gel (1 g) was placed on the donor compartment. A polyethersulfone membrane with 0.45 μm pore size was used as model membrane. An aliquot of 0.5 mL was taken from the receptor compartment at the predetermined time points and then replaced with the same volume of release medium to maintain sink conditions. The experiments were performed at constant temperature maintained at 32 °C. All samples were further analyzed spectrophotometrically (UV-1800, Shimadzu) at 258 nm.

## 3. Results and Discussion

kCR, kCR/MTX, kCR/βCD, and kCR/MTX/βCD were prepared and characterized with the aim of revealing the effect of the additives (MTX, βCD, and MTX/βCD inclusion complexes) on the physicochemical and pharmacological properties of the aforementioned gels designed for topical administration.

The preliminary tests allowed for the establishment of the optimal concentration of kCR. It was found that gels with a kCR content <1.25 wt.% undergo a gel–sol transition temperature below 35 °C (Figure S2I), thus limiting their topical application. On the other hand, gels with a kCR content ≥1.25 wt.% were stable at the physiological temperatures (Figure S2I), but their consequent high rigidity also restricted their topical use. Therefore, the gel with a kCR content of 1.25 wt.% was selected as the most suitable for the purpose of this study.

### 3.1. Linear Viscoelastic Properties

Rheological properties play an important role in the design of topical pharmaceutical formulations. In this work, the rheological characteristics of the gels were obtained under various regimes of shearing for the evaluation of MTX, βCD, and MTX/βCD inclusion complex regarding the structure of kCR gel.

**Figure 2:**
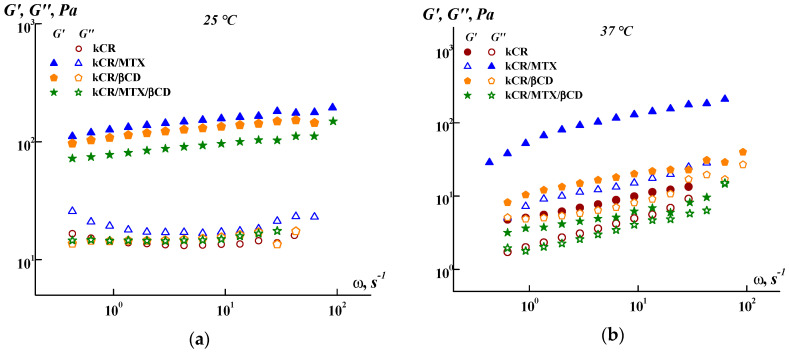
Frequency dependence of the storage (*G*′) and loss (*G*″) moduli at 25 °C (**a**) and 37 °C (**b**).

At first, the viscoelastic properties of the samples were measured in the guaranteed linear viscoelastic domain. Figure 2a displays the frequency sweep of the storage modulus (*G*′) and loss modulus (*G*″) for kCR, kCR/MTX, kCR/βCD, and kCR/MTX/βCD gels at 25 °C. The results show that *G*′ > *G*″ for all the systems. Moreover, both modules are very weakly dependent on the applied frequency. Such viscoelastic behavior is typical for strong gels [34]. It is believed that kCR is able to form gels with a three-dimensional network structure. In these systems, junction zones are the helical aggregates, which are formed via a conformational transition (caused by decrease of temperature) of random coils of the polymer to the double helices [35]. The junction zones in kCR gel can also be the aggregates of double helices containing 2–10 polysaccharide strands [36]. This particularity determines the formation of rigid kCR gels and distinguishes it from softer gels of iCR, for which the coil-to-double helix transition process shows second-order kinetics, indicating only dimerization [37].

As shown in Figure 2a, the additives used (MTX, βCD, or MTX/βCD inclusion complexes) slightly affect the *G*′ values, without influencing the *G*″ values at 25 °C. However, the effect of these additives on *G*′ and *G*″ becomes more pronounced at 37 °C (Figure 2b). This fact points out that the lability of the three-dimensional network of kCR is changed with the temperature rise and this promotes the interactions of kCR with MTX, βCD, or MTX/βCD.

It should be noted that the difference between the *G*′ values obtained for kCR/MTX gel at 25 °C and 37 °C is less than for kCR/βCD and kCR/MTX/βCD gels (Figure 2). This means that the MTX molecules are more capable of stabilizing the structure of kCR gel, making it less sensitive to the temperature rise. Such a stabilizing effect is apparently caused by a decrease in the mobility of the polymeric chains due to the interactions between MTX and kCR. MTX has carboxylic and amino groups in the structure (Figure 1), which are able to conduct ionization (pK_a1_ = 3.22, pK_a2_ = 4.53 and pK_a3_ = 5.62 [38]). kCR contains –OH and –SO_3_^−^ groups (Figure 1). Thus, binding of MTX with kCR can occur mainly through the electrostatic interactions, H-bonds formation, van der Waals forces, and hydrophobic interactions [39]. These multiple interactions were also observed between iCR and MTX [20]. It can be hypothesized that MTX incorporation both in junction zones and interjunction connections in kCR gel increases the stability of its structure and dramatically decreases the mobility of the biopolymer macromolecules.

Meanwhile, βCD also has a stabilizing effect on the kCR gel, as in Figure 2, even if it is less pronounced than with MTX. In a previous work, the authors confirmed the binding of CDs to a kCR via hydrogen bonding [40]. A rearrangement of random kCR coils in the presence of CDs, which lead to a more uniform distribution of kCR molecules in water, has been postulated according to the rheological data and SEM images [40,41]. It has been also demonstrated by Wang et al. [42] that hydroxypropyl-β-CD affected the kCR gelation mainly by facilitating the ordering of kCR coils and preventing the aggregation of kCR helices. Hydroxypropylated β-CD enabled gel formation as well as gel melting at a slightly higher temperature, compared with the “empty” kCR gel [42].

Conversely, the kCR/MTX/βCD gel is characterized by lower *G*′ values than the pure kCR gel (Figure 2). This effect is pronounced at 37 °C. Moreover, the kCR/MTX/βCD gel demonstrates the smallest gap between *G*′ and *G*″ at 37 °C, confirming the gel structure in the presence of MTX/βCD inclusion complexes. Apparently, the presence of MTX/βCD complexes results in the formation of a more labile structure of the kCR gel. It is important to note that the kCR/MTX/βCD gel still has *G*′ > *G*″ at 37 °C, retaining the gel at human body temperature.

### 3.2. Steady Flow Properties

**Figure 3:**
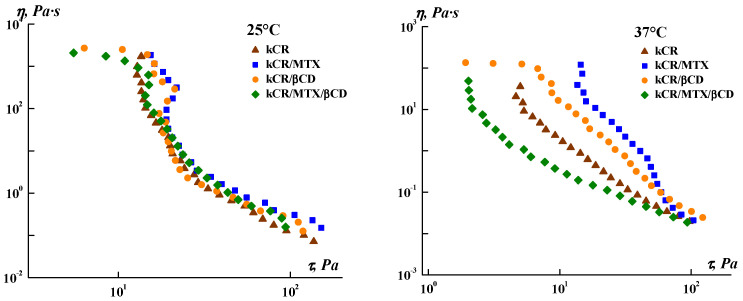
Dependence of the apparent viscosity on the shear stress for the gels under study.

The possibility of application of the topical gels is determined by their flow behavior. Therefore, we explored the rheological properties of the gels under steady shear. Figure 3 displays the flow viscosity of the samples versus the shear stress. One can see a similarity in the behavior of all the gels tested at 25 °C, whereas the noticeable difference in the rheological behavior of the samples was observed at 37 °C. Addition of MTX, βCD, or MTX/βCD inclusion complexes to kCR gel does not change its non-Newtonian pseudoplastic behavior, but essentially affects its yield shear stress and apparent viscosity.

The influence of the additives on the rheological behavior of kCR gels at 37 °C (Figure S3I) was assessed by using several mathematical models:

Herschel–Bulkley model

*τ* = *τ*_0_ + *kγ^n^* (1)

Bingham model

*τ* = *τ*_0_ + *η_p_γ* (2)

Casson model

*τ*^0.5^ = *τ*_0_^0.5^ + (*η_p_γ*)^0.5^ (3)

where *τ* is the shear stress (Pa); *τ*_0_ is the yield shear stress (Pa); *k* is the consistency coefficient (Pa·s^n^); *γ* is the shear rate (s^−1^); *η_p_* is plastic viscosity (Pa·s); *n* is the flow coefficient. The rheological parameters and correlation coefficients (*R*^2^) specific to these models are listed in Table 1.

As can be seen from Table 1, the Herschel–Bulkley equation provides the most appropriate fit for all the gels (*R*^2^ were the highest). The yield shear stress (*τ*_0_) of a material is defined as the minimum stress needed to start the flow. The consistency coefficient (*k*) is close to the average viscosity of materials in the entire shear rate range [43]. According to the obtained results, the addition of MTX or βCD increased the values of *τ*_0_ and *k*, and the influence of MTX is more significant. On the contrary, minimum values of *τ*_0_ and *k* were obtained for kCR/MTX/βCD gel. Thus, kCR/MTX/βCD gel is more spreadable than the other gels under consideration.

The flow index values (*n* < 1) confirm that all systems are pseudoplastic fluids. The *n* values are decreased with the addition of MTX or βCD to kCR gel and they are increased for the kCR/MTX/βCD system. The obtained data indicate that MTX and βCD strengthen the gel structure; however, after overcoming the yield stress, the structure breaks more easily under the action of high-speed shear. Inclusion complexes MTX/βCD, on the contrary, reduce both the gel strength and sensitivity of the gel structure to shear deformations imposed on the samples in this experiment. It should be noted that the *k* value for the kCR/MTX/βCD gel is lower than that of the kCR/MTX sample, so the former will be easier to apply on the skin or tissues.

### 3.3. SEM Analysis

**Figure 4:**
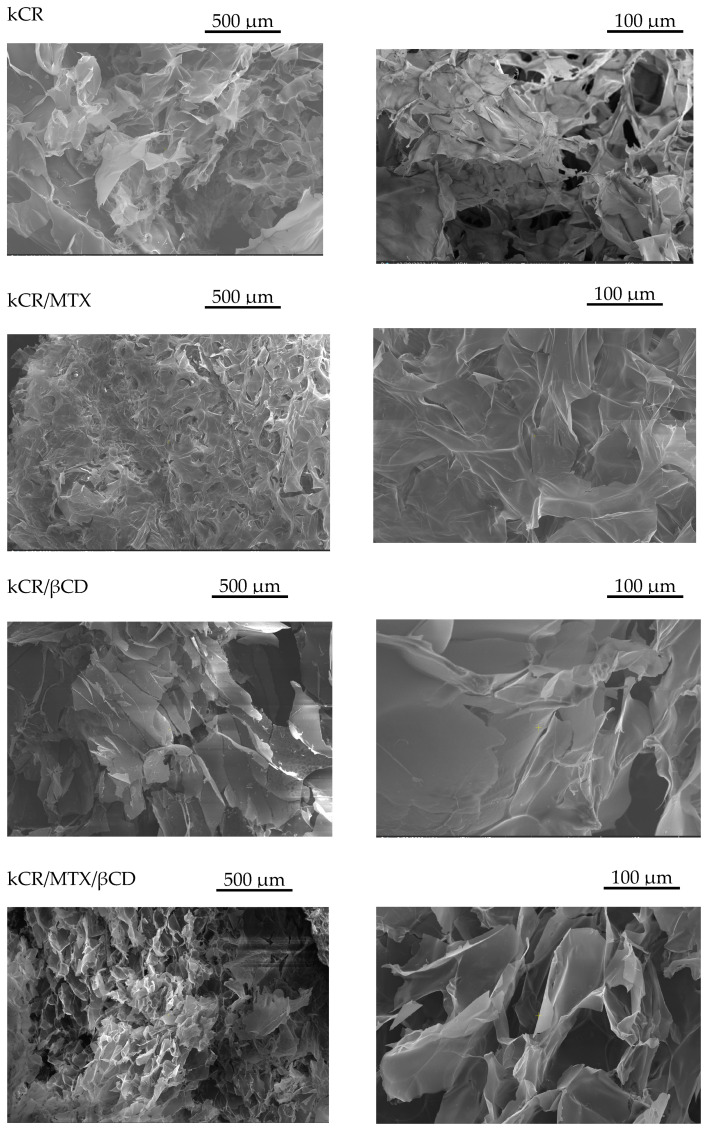
SEM images of freeze-dried gels under study.

SEM analysis was performed to study the surface morphology of the freeze-dried gels. The micrographs reported in Figure 4 demonstrate that the gels hold a porous structure, a denser and more even texture with smaller pore sizes, which could be due to the interactions between the kCR helixes and MTX. In the case of kCR/βCD gel, the structure is non-uniform with a lower quantity of pores as compared with the pure kCR sample. A similar effect of βCD on the kCR gel has been reported by Yuan C. et al. [40]. Larger pores have been observed in kCR/MTX/βCD compared to the pure kCR gel.

### 3.4. ^1^H NMR and FTIR Spectroscopy

**Figure 5:**
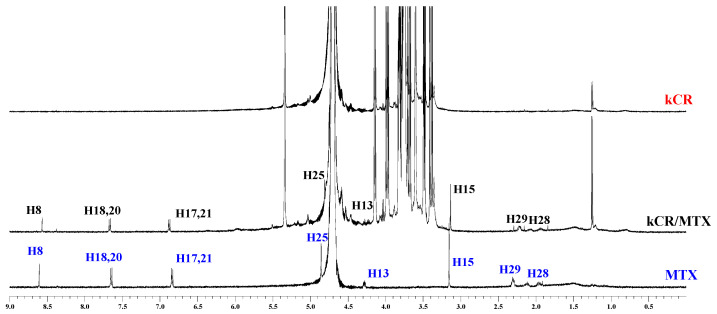
^1^H NMR spectra of MTX (blue color), hydrogels of kCR (red color) and kCR/MTX (black color) at 25 °C.

To reveal the nature of the interactions between kCR and the additives (MTX and βCD) in the gels, the ^1^H NMR experiments were carried out. ^1^H NMR spectra of MTX in its pure form and incorporated in the kCR gel are shown in Figure 5. As is evident from the comparative analysis of the ^1^H NMR spectra, the signals from MTX protons H25, H28, and H29 located near the polar amino and carboxylic groups of MTX (Figure 1) are upfield shifted. It seems that the polar fragment of the MTX molecule participates in the binding with kCR. Unfortunately, the ^1^H NMR spectra of kCR gels with βCD were not suitable for analysis due to the overlapping of their main characteristic signals.

**Figure 6:**
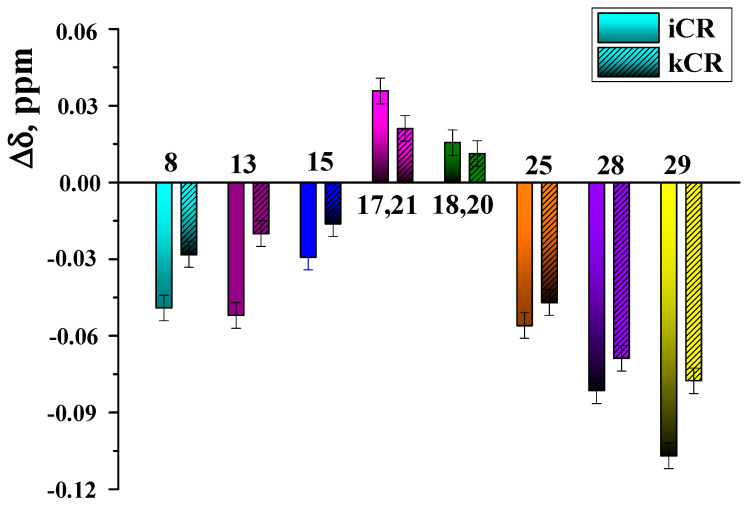
Chemical shift changes of MTX protons induced by the presence of kCR (0.4 wt.%) and iCR (4 wt.%) in solution (D_2_O, 25 °C).

Binding of MTX with kCR was additionally studied in solution. Chemical shift changes of MTX protons induced by interactions with kCR were measured and given in Figure 6. As one can see, more pronounced changes were observed for protons H25, H28 and H29 located near polar side groups of MTX (Figure 1). More probably, these polar groups are involved in the binding with kCR via hydrogen bonding.

It was interesting to evaluate the role of –SO_3_^−^ groups in CR molecule in the binding with MTX. To this aim, the binding affinity of MTX to kCR and iCR [20] in solution was compared. As follows from the obtained Δ*δ* values (Figure 6), more significant chemical shift changes of MTX protons were observed in the presence of 0.4 wt.% iCR. iCR has two sulfate groups per disaccharide repeating unit (Figure 1). Consequently, the number of binding sites with MTX is higher in iCR compared with kCR having only one –SO_3_^−^ group per disaccharide unit.

Interactions of kCR with MTX and βCD in solid state were studied by FTIR spectroscopy. To this end, FTIR spectra of freeze-dried gels under study were recorded and analyzed (Figure S4I). As it was observed, main characteristic bands of pure kCR, MTX and βCD were remained unchanged in the spectra of the gels. This fact points out the absence of chemical interaction between kCR network and additives (MTX and βCD) in the solid state.

### 3.5. DLS

**Figure 7:**
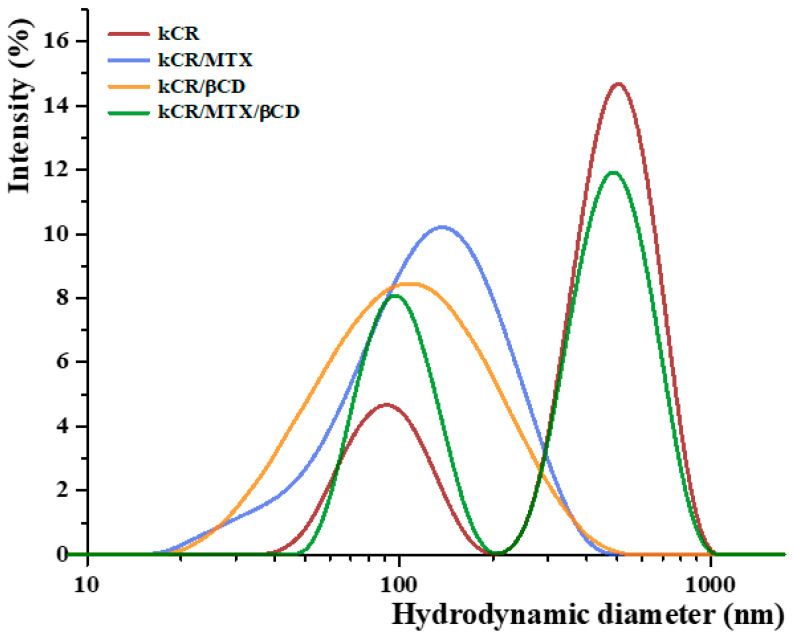
Size distribution spectra of the samples under study at 25 °C.

DLS is the most popular method for determining polymer sizes in solution. DLS measurements of the dilute kCR solutions without and with the additives under study were performed with the aim of revealing the influence of MTX, βCD, or MTX/βCD inclusion complexes on the aggregation behavior and conformation of kCR macromolecules. The obtained results are shown in Figure 7. As one can see, pure kCR solution has a bimodal intensity size distribution with the peaks corresponding to hydrodanamic diameters of 91 nm and 503 nm. A similar size distribution for kCR solution has been previously reported by Antonov et al. [44]. The authors pointed out that these two peaks probably originate from kCR macromolecules (coils) and their aggregates.

As one can see from Figure 7, addition of MTX (3 × 10^−4^ M) or βCD (0.009 M) to kCR solution (0.01 wt.%) causes the disappearence of the peak corresponding to the existence of large aggregates. Upon addition of βCD to kCR solution, the tendency of kCR to aggregate is reduced, and one broad peak with the mode at 105 nm is observed (Figure 7). A similar peak corresponding to a hydrodynamic diameter of 136 nm appears for kCR solution with MTX content. Most likely, the revealed changes in the particle size distribution are due to the conformational rearrangement of biopolymer macromolecules caused by their possible interactions with the additives. A broadening of the peak induced by decreasing temperature of kCR solution has been reported by Abad et al. [45]. This phenomenon has been assigned to the appearance of particles with higher rigidity due to the coil-to-helix transition of kCR. Apparently, MTX and βCD can interact with kCR via multiple noncovalent interactions, promoting the helical conformation of the biopolymer.

The bimodal particle size distribution was detected for kCR solution in the presence of MTX/βCD inclusion complexes (Figure 7). It seems that MTX/βCD complexes have no considerable influence on the kCR aggregation. It is more likely that βCD and MTX have higher affinity to interact with each other than with kCR. Therefore, the behavior of kCR in the presence of the complexes is similar to the behavior of pure kCR. However, a decrease in the intensity of the peak corresponding to large particles and an increase in the intensity of peak representing the individual kCR coils should be noted. This fact can be explained by the ability of the complexes to prevent aggregation of the kCR coils.

### 3.6. Release Study

**Figure 8:**
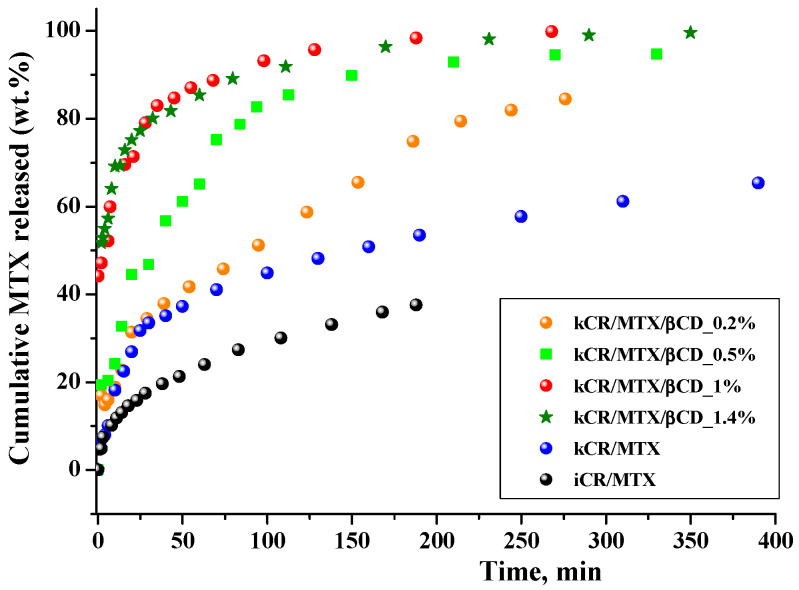
Release profiles of MTX from CR gels (1.25 wt.%) in phosphate buffer (pH = 7.4) at 37 °C.

The ability of kCR gels to release the MTX was investigated in vitro in phosphate buffer at 37 °C. Figure 8 shows the release profiles of MTX from hydrogels. The release rate from kCR/MTX gel is relatively low, −65 wt.% of the drug during 6 h. It can be seen for comparison that kCR and iCR gels of the same composition released MTX, reaching 50 wt.% and 35 wt.%, respectively, after 2.5 h. The faster release from kCR gel can be explained by the revealed weaker binding of MTX with kCR than with iCR.

βCD incorporation in the kCR gels induces a high release of MTX. For instance, full MTX release (100 wt.%) is achieved in 4 h in the presence of 1 wt.% βCD. The obtained results are in accordance with the rheological properties of these gels. Figure 3 and Table 1 show that kCR/MTX/βCD gel is less viscous and more spreadable than kCR/MTX at 37 °C. Most likely, it facilitates the diffusion of MTX. Moreover, the additional reason for the observed phenomenon is the inclusion complex formation occuring between MTX and βCD in solution [20,32]. The MTX content is larger in kCR/MTX/βCD than in kCR/MTX. Consequently, the higher concentration gradient determines the faster release of MTX from kCR/MTX/βCD than kCR/MTX gel. However, the concentration gradient is not a key factor governing the release rate. To confirm this, we prepared kCR/MTX/βCD and kCR/MTX gels with the same MTX concentration and found that the release from the first of the mentioned gels proceeds faster (Figure S5I). Moreover, we believe that the affinity of MTX is stronger to βCD than to kCR. This assumption is supported by the opposite rheological behavior of kCR/MTX and kCR/MTX/βCD gels at 37 °C (Figure 3). Inclusion complexes of CDs are water-soluble due to the availability of the external –OH groups surrounding the macrocyclic cavity. Thus, MTX inserted into βCD displays higher affinity to an aqueous environment compared with the uncomplexed MTX.

The MTX release rate increases with βCD content in kCR/MTX/βCD gel from 0.2 wt.% to 1.0 wt.%. However, further increase in βCD concentration up to 1.4 wt.% has no influence on the MTX release rate. This behavior is in accordance with the increase in the concentration of the inclusion complexes, which are formed between MTX and βCD in solution. The dependence of the inclusion complexes concentration on βCD concentration in solution was obtained considering the previously determined binding constant (K = 736 M^−1^ [20]), and given in Figure S6I. The concentration of the inclusion complexes increases with a βCD amount up to 1 wt.%, and after that, it is not changed. This fact could be taken into account for prediction of the MTX release rate from the gels with the variable βCD content.

The release of MTX from kCR gels of different composition was mathematically described using different kinetic models such as:

zero-order model

*Q_t_*/*Q*_∞_ = *K*_0_*t* (4)

first-order model

*log Q_t_*/*Q*_∞_ = −*K*_1_·*t*/2.303 (5)

Higuchi model

*Q_t_*/*Q*_∞_ = *K_H_*·*t*^1/2^ (6)

Hixson–Crowell model

*Q*_∞_^1/3^ − *Q_t_*^1/3^ = *K_HC_*·*t* (7)

Korsemeyer–Peppas model

*Q_t_*/*Q*_∞_ = *K_KP_*·*t^n^* (8)

where *Q_t_*/*Q_∞_* represents the fractional drug release; *K*_0_, *K*_1_, *K_H_*, *K_HC_*, and *K_KP_* are the kinetic constants of each mathematical model; *n* is release exponent. The best fitting was revealed considering the values of the correlation coefficients (*R*^2^) summarized in Table 2. The Korsemeyer–Peppas model was the most appropriate model to predict the MTX release from the gel, as indicated by the highest *R*^2^. In this model, the *n* value is used to determine the release mechanism. Generally, the *n* values below 0.45 correspond to Fickian diffusion, while values between 0.45 and 0.89 reveal anomalous diffusion (non-Fickian) [46]. Based on Table 2, *n* < 0.45 values indicate the release of MTX from all gels under study is Fickian diffusion. The presence of βCD in the gels does not affect the mode of MTX release.

### 3.7. Permeation Study

**Figure 9:**
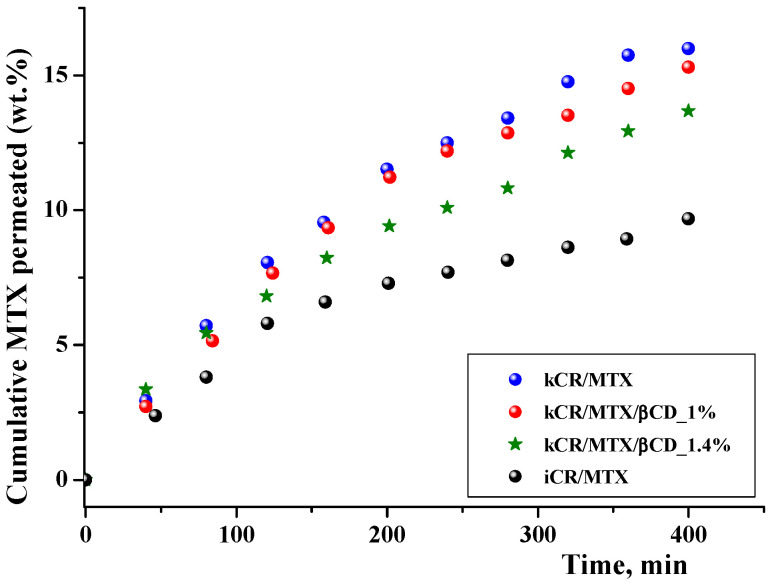
In vitro permeation profiles of MTX from gels on the basis of kCR and iCR (1.25 wt.%) at 32 °C.

Prediction of membrane permeability is necessary in the development of pharmaceutical formulations. MTX permeation through an artificial polyethersulfone microporous membrane (0.45 μm) that mimics the skin [47,48] was studied. It was found that the cumulative amount of free MTX released from kCR/MTX gel and passed through the membrane during 6 h was 16 wt.%. Complexation with βCD increases the MTX release rate, as is shown in Figure 8, but at the same time, reduces MTX permeation (Figure 9). It was demonstrated by means of ^1^H NMR spectroscopy that inclusion complexes of MTX with βCD are able to pass through the model polyethersulfone membrane. However, in comparison with the free MTX, the permeation rate of the inclusion complexes is lower due to their larger size. Permeation is governed by the stability constant of the complexes of MTX with βCD and decreases with the rise of βCD concentration (Figure 9). Therefore, gels of the basis of kCR provided the sustained release of MTX.

Significant differences in the permeation profiles of MTX from kCR and iCR were observed (Figure 9). This is in accordance with the faster release of MTX from kCR gel (Figure 8) and revealed the weaker binding affinity of MTX to kCR (Figure 6).

The area under the plasma drug concentration–time curve (AUC) obtained from Figure 9 can be used to predict in vivo AUC. Calculated values of AUC_0–6.7h_ are given in Table 2SI. This attempt is based on a linear in vivo–in vitro correlation that has been found by M. Klitgaard et al. [49] when comparing the area under the in vitro drug permeation–time curve to the AUC of the plasma concentration–time profile obtained from the in vivo study. Based on these results, the permeation method was proposed as a promising tool for estimating the in vivo performance.

## 4. Conclusions

Gels based on biocompatible kCR have been easily prepared and used for MTX entrapment. MTX concentration in the gel can be increased by means of βCD, which forms stable inclusion complexes with MTX. Moreover, stabilization of MTX in the gel can be achieved due to the inclusion complex formation with βCD. It was demonstrated that the addition of βCD to the gel formulations has no significant effects on the viscoelastic properties of the gels; meanwhile, it affects the MTX release from the gels. The MTX release profiles from kCR/MTX and kCR/MTX/βCD gels with variable βCD content were obtained and mathematically analyzed. It was found that MTX release from all gels follows Fickian diffusion, which can be controlled by increasing the βCD concentration in the formulation. Various behaviors of kCR/MTX and iCR/MTX gels were demonstrated and explained by the different binding affinity of MTX to CRs, having one and two –SO_3_^−^ groups.

## Figures and Tables

**Table 1:** Estimated rheological model constants and statistical parameters *.

Gel	Herschel-Bulkley Model				Bingham Model			Casson Model		
	*τ*_0_, Pa	*k*, Pa·s^n^	*n*	*R* ^2^	*τ*_0_, Pa	*η_p_*, Pa·s	*R* ^2^	*τ*_0_, Pa	*η_p_*, Pa·s	*R* ^2^
kCR	2.1 ± 0.5	3.8 ± 0.3	0.37± 0.01	0.99	12 ± 2	0.020 ± 0.001	0.82	2.4 ± 0.2	0.12 ± 0.01	0.84
kCR/MTX	6.4 ± 3.7	13.6 ± 3.7	0.21± 0.03	0.95	24 ± 3	0.019 ± 0.002	0.63	4.1 ± 0.3	0.10 ± 0.01	0.64
kCR/βCD	2.2 ± 1.0	8.9 ± 0.8	0.29± 0.01	0.99	17 ± 3	0.025 ± 0.002	0.77	2.8 ± 0.3	0.14 ± 0.01	0.76
kCR/MTX/βCD	0.7 ± 0.2	2.0 ± 0.1	0.45± 0.01	0.99	7 ± 2	0.026 ± 0.001	0.87	1.8 ± 0.1	0.13 ± 0.01	0.91

*—experimental data for 37 °C.

**Table 2:** Modeling of MTX release behavior from kCR gels of different composition.

Model	*R* ^2^				
	kCR/MTX	kCR/MTX/βCD__0.2%_	kCR/MTX/βCD__0.5%_	iCR/MTX/βCD__1%_	iCR/MTX/βCD__1.4%_
Zero-order	0.735	0.939	0.619	0.565	0.662
First-order	0.455	0.802	0.473	0.486	0.575
Higuchi	0.832	0.982	0.836	0.803	0.850
Hixson-Crowell	0.549	0.859	0.5242	0.512	0.604
Korsemeyer-Peppas	0.931 (*n* = 0.44)	0.977 (*n* = 0.41)	0.910 (*n* = 0.41)	0.934 (*n* = 0.08)	0.962 (*n* = 0.10)

## Data Availability

Data is contained within the article or Supplementary Materials.

## Supplementary Materials

The following supporting information can be downloaded at: https://www.mdpi.com/article/10.3390/pharmaceutics15092244/s1, Figure S1I: Calibration curve for spectrophotometric determination of MTX in phosphate buffer; Figure S2I: Temperature dependence of the storage (*G*′) and loss (*G*″) moduli for the kCR gels (a −1 wt.% kCR; b −1.25 wt.% kCR); Figure S3I: Flow curves for gels under study; Figure S4I: FTIR spectra of freeze-dried gels under study; Figure S5I: Release profiles of MTX from kCR gels (1.25 wt.%) in phosphate buffer (pH = 7.4) at 37 °C (concentration of MTX in the gels is the same); Figure S6I: Concentration of the inclusion complexes of MTX with βCD formed in solution *versus* βCD concentration; Table S1I: Content of the components in gels on the basis of kCR; Table S2I: AUC_0–6.7h_ in vitro of permeated MTX from the gels under study.
