# Supplementary material for: β-Cyclodextrin Modified Hydrogels of Kappa-Carrageenan for Methotrexate Delivery
Source: Pharmaceutics. 2023 Aug 30;15(9):2244. doi: 10.3390/pharmaceutics15092244 (PMC10535384; doi:10.3390/pharmaceutics15092244)
Supplement: Supplementary file 1 [file pharmaceutics-15-02244-s001.zip › pharmaceutics-2529287-supplementary.pdf]

pharmaceutics

Supplementary Material

β-Cyclodextrin modified hydrogels of kappa-carrageenan for methotrexate delivery

Maria Nikitina, Nataliya Kochkina, Marianna Arinina, Valery Kulichikhin and Irina Terekhova

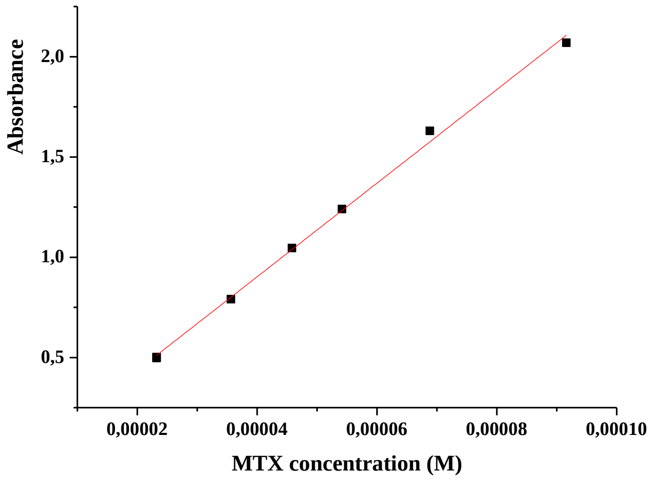

Figure 1. SI. Calibration curve for spectrophotometric determination of MTX in phosphate buffer.

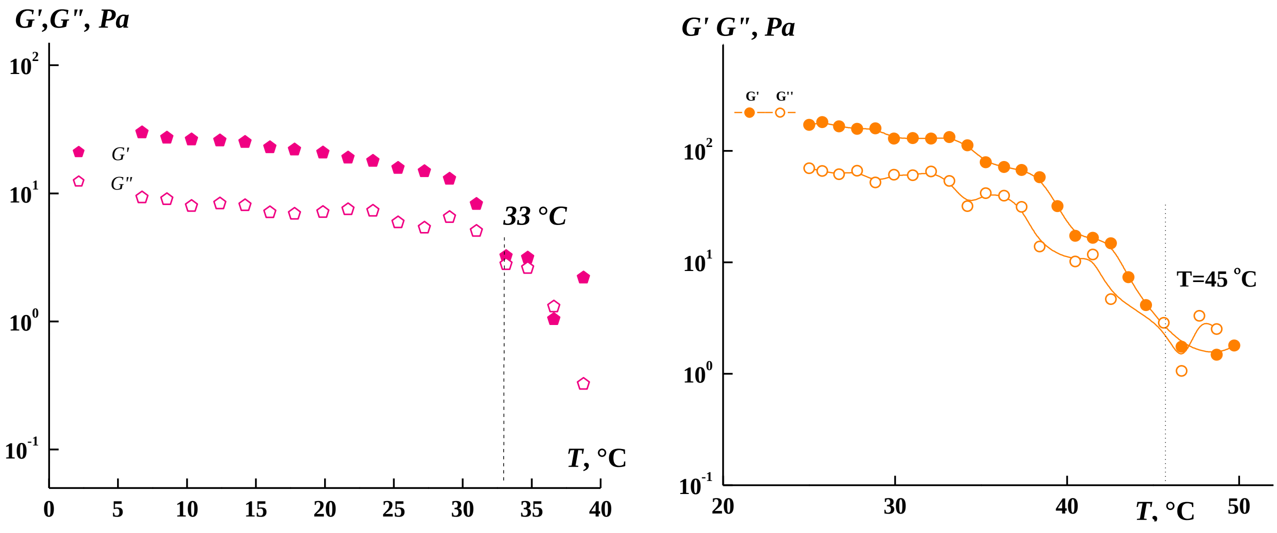

Figure 2. SI. Temperature dependence of the storage (G′) and loss (G′′) moduli for the kCR gels (a – 1 wt.% kCR; b – 1.25 wt.% kCR).

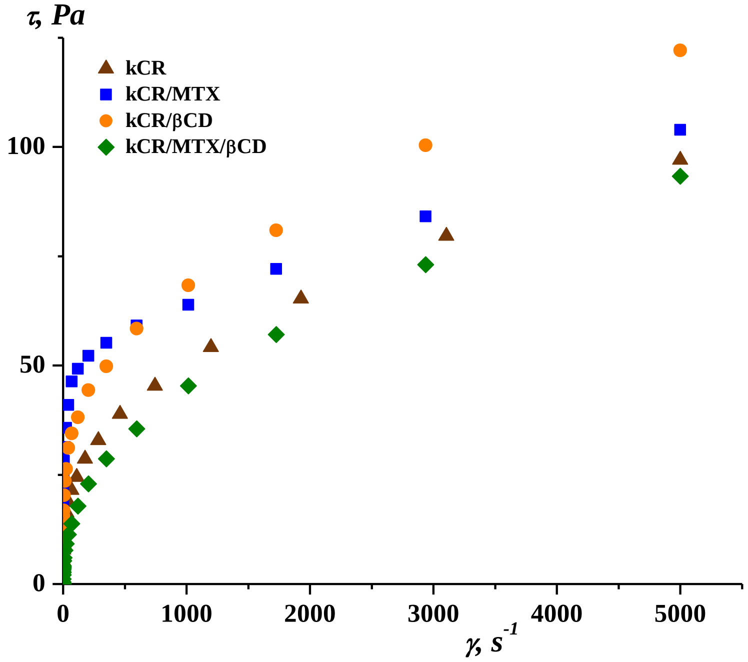

Figure 3. SI. Flow curves for gels under study.

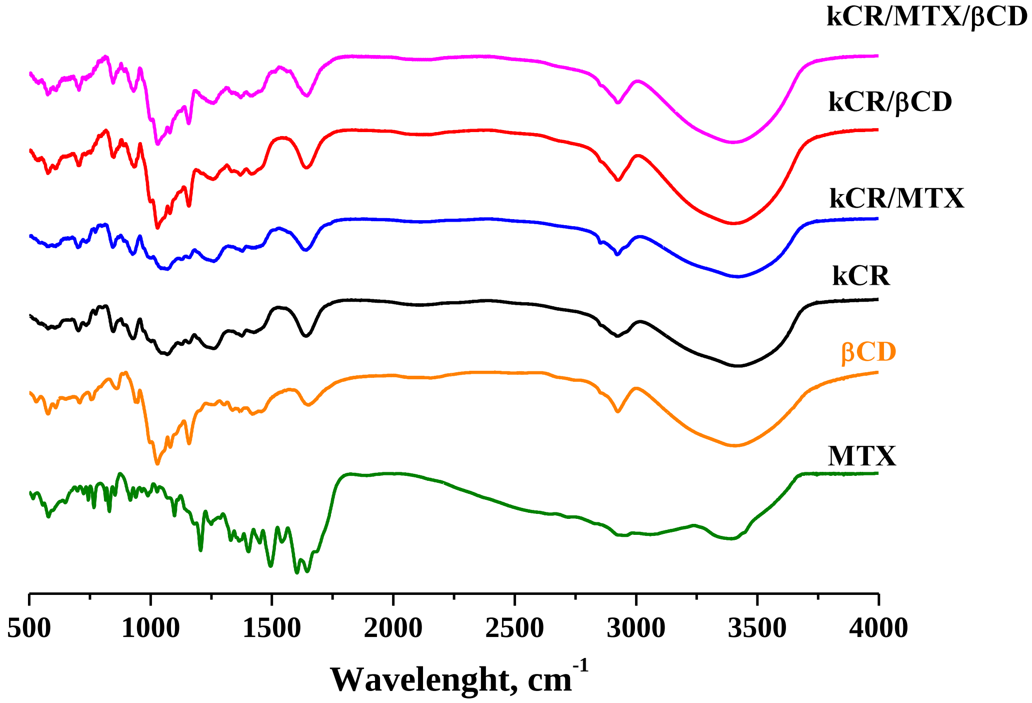

Figure 4. SI. FTIR spectra of freeze-dried gels under study.

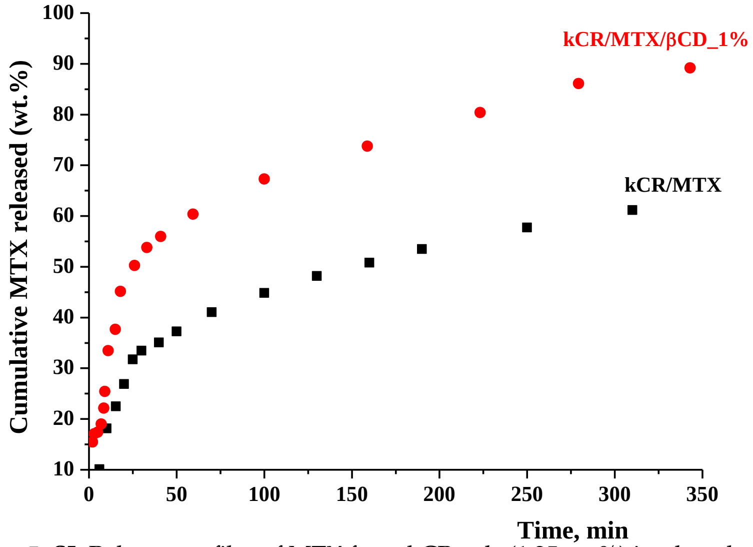

Figure 5. SI. Release profiles of MTX from kCR gels (1.25 wt.%) in phosphate buffer (pH=7.4) at 37 °C (concentration of MTX in the gels is the same).

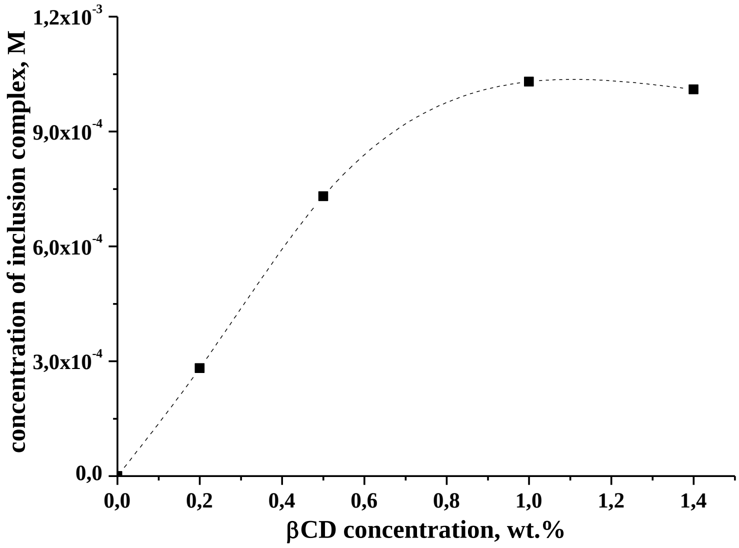

Figure 6. SI. Concentration of the inclusion complexes of MTX with βCD formed in solution *versus* βCD concentration.

Table 1. SI. Content of the components in gels on the basis of kCR.

Gel	MTX, M	βCD, wt.%	kCR, wt.%
kCR/MTX	$2.0 \cdot 10^{-4}$	0	1.25
kCR/MTX/βCD_$_{0.2\%}$	$5.5 \cdot 10^{-4}$	0.2	1.25
kCR/MTX/βCD_$_{0.5\%}$	$1.0 \cdot 10^{-3}$	0.5	1.25
kCR/MTX/βCD_$_{1\%}$	$1.20 \cdot 10^{-3}$	1.0	1.25
kCR/MTX/βCD_$_{1.4\%}$	$1.13 \cdot 10^{-3}$	1.4	1.25

Table 2. SI. $AUC_{0\text{-}6.7h}$ *in vitro* of permeated MTX from the gels under study.

Gel	$AUC_{0\text{-}6.7h}$ (wt.%/min)	$AUC_{0\text{-}6.7h}$(gel)/$AUC_{0\text{-}6.7h}$(kCR/MTX)
kCR/MTX	4090	1
kCR/MTX/βCD_$_{1\%}$	3850	0.94
kCR/MTX/βCD_$_{1.4\%}$	3441	0.84
iCR/MTX	2552	0.62
